# Modulatory effects of BPC 157 on vasomotor tone and the activation of Src-Caveolin-1-endothelial nitric oxide synthase pathway

**DOI:** 10.1038/s41598-020-74022-y

**Published:** 2020-10-13

**Authors:** Ming-Jer Hsieh, Cheng-Hung Lee, Ho-Yen Chueh, Gwo-Jyh Chang, Hsiu-Yun Huang, Yuling Lin, Jong-Hwei S. Pang

**Affiliations:** 1 Graduate Institute of Clinical Medical Sciences, College of Medicine, Chang Gung University, 259 Wen-Hwa 1st Road, Kwei-Shan, Tao-Yuan City, Taiwan, ROC; 2 Division of Cardiology, Department of Internal Medicine, Chang Gung Memorial Hospital-Linkou, Chang Gung University, Tao-Yuan City, Taiwan, ROC; 3 Department of Obstetrics and Gynecology, Chang Gung Memorial Hospital-Linkou, Chang Gung University, Tao-Yuan City, Taiwan, ROC; 4 Department of Physical Medicine and Rehabilitation, Chang Gung Memorial Hospital, Kwei-Shan, Tao-Yuan City, Taiwan, ROC

**Keywords:** Drug discovery, Molecular biology, Cardiology, Molecular medicine

## Abstract

BPC 157-activated endothelial nitric oxide synthase (eNOS) is associated with tissue repair and angiogenesis as reported in previous studies. However, how BPC 157 regulates the vasomotor tone and intracellular Src-Caveolin-1 (Cav-1)-eNOS signaling is not yet clear. The present study demonstrated a concentration-dependent vasodilation effect of BPC 157 in isolated rat aorta. Attenuation of this vasodilation effect in the absence of endothelium suggested an endothelium-dependent vasodilation effect of BPC 157. Although slightly increased vasorelaxation in aorta without endothelium was noticed at high concentration of BPC 157, there was no direct relaxation effect on three-dimensional model made of vascular smooth muscle cells. The vasodilation effect of BPC 157 was nitric oxide mediated because the addition of L-NAME or hemoglobin inhibited the vasodilation of aorta. Nitric oxide generation was induced by BPC 157 as detected by intracellular DFA-FM DA labeling which was capable of promoting the migration of vascular endothelial cells. BPC 157 enhanced the phosphorylation of Src, Cav-1 and eNOS which was abolished by pretreatment with Src inhibitor, confirming the upstream role of Src in this signal pathway. Activation of eNOS required the released binding with Cav-1 in advance. Co-immunoprecipitation analysis revealed that BPC 157 could reduce the binding between Cav-1 and eNOS. Together, the present study demonstrates that BPC 157 can modulate the vasomotor tone of an isolated aorta in a concentration- and nitric oxide-dependent manner. BPC 157 can induce nitric oxide generation likely through the activation of Src-Cav-1-eNOS pathway.

## Introduction

Pentadecapeptide BPC 157 is known to possess therapeutic efficacy on variable tissue healing and angiogenesis that is considered through the activation of nitric oxide system as reported in previous studies^1–6^. Since the first demonstration of nitric oxide generation in gastric mucosa which contributed to the antiulcer effect of BPC 157 in gastric lesion assay by Sikiric et al.^5^, following studies analyzing the influence by treating together or alone with nitric oxide inhibitor, *N*^ω^-nitro-l-arginine methyl ester (L-NAME) or nitric oxide precursor, L-arginine all showed that the nitric oxide modulation is involved in the healing effect of BPC 157 in different tissue injuries. A considerable number of evidences provided by Sikiric et al. further demonstrated the modulatory role of BPC 157 on nitric oxide generation^7–11^. Our previous study reveals that BPC 157 can markedly promote the expression of vascular endothelial growth factor VEGF receptor 2 (VEGFR2) and angiogenesis in ischemic hind limb^6^. BPC 157 accelerated the blood flow recovery in ischemic hind limb simply through angiogenesis, since there was no significant difference of the blood flow or pressure in tails between the control and BPC 157 groups. In the same study, the phosphorylation of endothelial isoform of nitric oxide synthase (eNOS) in vascular endothelial cells is found to be quickly induced by BPC 157 within 30 min. Vessel relaxation is one of the various biological functions of nitric oxide^12^. Therefore, it is interesting to further investigate whether the activation of eNOS by BPC 157 could induce nitric oxide generation in vessels and how the vessel tone could possibly be regulated by BPC 157.

The eNOS plays a major role in vascular homeostasis through release of nitric oxide^13^. The activity of eNOS is tightly regulated by multiple intracellular process including posttranslational modification, phosphorylation and protein–protein interactions^14,15^. Within vascular endothelial cell, a group of proteins include calmodulin, heat shock protein 90 and protein kinase B/AKT have identified as positive regulators of eNOS activity^16^. In addition, Caveolin-1 (Cav-1), a 21-kDa integral plasma membrane protein is a well-known negative regulator of eNOS activity in vascular endothelial cells^17–22^. Cav-1 directly interacts with eNOS and holds eNOS on an inactive status in caveolae. Upon stimulation by agents such as VEGF, the binding of eNOS and Cav-1 is released, eNOS associates with calmodulin, and then subsequently phosphorylated by heat shock protein 90 and AKT^23^. Cav-1 has also been proved to modulate vascular tone in animal models. In addition, Src-dependent Cav-1 phosphorylation is a crucial process mediating caveolae-associated endocytosis and eNOS activation in vascular endothelial cells^24,25^. However, effects of BPC 157 on vascular tone and intracellular Src-Cav-1-eNOS signaling are still not understood. Hence there were two main goals of this study. First, we investigated effect of BPC 157 on vasomotor tone of blood vessel to study if it was in an endothelium- or nitric oxide-dependent manner. Second, we explored whether BPC 157 could modulate the protein–protein interaction between Cav-1 and eNOS, and the Src-Cav-1-eNOS signaling activation.

## Methods and materials

### Reagents

Pentadecapeptide BPC 157 (GEPPPGKPADDAGLV, M.W.1419) was synthesized by Kelowna International Scientific Inc. (Taiwan, ROC). Phenylephrine (PE), acetylcholine (ACh), L-NAME, hemoglobin and sodium nitroprusside (SNP) were purchased from Sigma Chemical Company (St. Louis, MO, USA). All the drugs were prepared by dissolving in ddH_2_O and sterilized before use.

### Animal and tissue

Adult male SD rats, weight 250–300 g were purchased from National Laboratory Animal Center (Taipei, Taiwan, ROC). The animals were sacrificed by cervical dislocation under anesthesia with an i.p. injection of 50 mg/kg sodium pentobarbital (Sigma), and thoracic aorta was carefully dissected and maintained at 37 °C in Krebs solution containing 118.2 mM NaCl, 4.7 mM KCl, 1.2 mM MgSO_4_, 25 mM NaHCO_3_, 1.2 mM KH_2_PO_4_, 1.9 mM CaCl_2_ and 11.7 mM dextrose (pH 7.4, 95% O_2_, 5% CO_2_). All methods were approved by Chang Gung University Institutional Animal Care and Use Committee (Approval No. CGU15-001) and carried out in accordance with relevant guidelines and regulations.

### Vascular reactivity assessment

The thoracic aorta was dissected into rings with 4–5 mm in width. In endothelium-denuded study, a cotton stick was used to gently remove the endothelium by rubbing the intimal surface of vessel. The rings were mounted in an isolated organ chamber containing Krebs solution (pH 7.4) at 37 °C and kept constant aerating with 95% O_2_/5% CO_2_. Changes of isometric tension were measured with force transducer (FORT 10, WPI, Sarasota, FL, USA). The ring tension was manually adjusted to 1.0 g and equilibrated for 30 min. The plasma PE and ACh were used for the induction of vasoconstriction and vasorelaxation, respectively. The lack of relaxation effects by 3 μM ACh in rings pre-contracted with 3 μM PE confirmed the complete endothelium removal. Vasorelaxation at least 60% by 3 μM ACh in rings pre-contracted with 3 μM PE was considered endothelium intact. Then, each ring was washed and re-equilibrated for 60 min. Aortic rings were then pre-contracted with 3 μM PE. After a stable plateau was reached, BPC 157 was added in a cumulative manner to the bath. Relaxation induced by each concentration of BPC 157 was measured after the response reached steady-state, and the value was expressed as a percentage of the initial vasoconstrictor-induced tone. To examine the nitric oxide-dependency in this system, the rings were pretreated with 0.03 mM L-NAME, an eNOS inhibitor or 10 μM hemoglobin, a nitric oxide scavenger. Pretreatment continued for 20 min before PE was added. After sustained tone was established, BPC 157 was added cumulatively to the bath.

### Human umbilical vein endothelial cells (HUVECs) culture

HUVECs purchased from BCRC (Hsinchu, Taiwan, ROC) were grown in M199 medium supplemented with 16% fetal bovine serum and 20% EGM-2 (Clonetics, USA). Cells were passaged at confluence with 0.05% trypsin and maintained in a 37 °C incubator with humidified atmosphere of 5% CO_2_ and 95% air. Cells were passaged 3–5 times prior to be used in experiments.

### Three-dimensional (3-D) model of vascular smooth muscle cells

Rat vascular smooth muscle cells were maintained in high glucose DMEM supplemented with 10% fetal bovine serum. The 3-D cell model was made by culturing cells (2 × 10^5^–1.0 × 10^6^) with high glucose DMEM supplemented with 30% bovine collagen solution overnight. Cell disc formed in the 6 cm dish was ready for the test of contraction or relaxation by treating with different reagents including 1.0 μg/ml BPC 157, 100 μg/ml BPC 157 and 200 μM nitric oxide donor SNP. After incubation in indicated medium for 24 h, the diameter of each cell disc before and after incubation was measured and ratio was calculated.

### Qualitative assessment of nitric oxide production by fluorescent dye 3-Amino-4-aminomethyl-2′,7′-difluorescein, diacetate (DAF-FM DA, Sigma)

HUVECs grown in M199 based culture medium was stimulated with 1.0 μg/ml BPC 157 for 30 min to induce the nitric oxide generation. Cells were labeled with 2 μM fluorescent nitric oxide indicator DFA-FM DA and fluorescence was detected by fluorescent microscope with a standard fluorescein bandpass filter (FITC, 500–555 nm). The final fluorescence signal was normalized by cell number.

### Transwell migration assay

Transwell filters (Costar, Corning, Cambridge, MA, USA) with 8.0 μm pores were used for the migration assay. HUVECs were seeded at a density of 1 × 10^5^ cells per filter. The inner chamber was filled with 200 μl M199, and the outer chamber was filled with 600 μl M199 containing 1% FBS and 1 μg/ml BPC 157. Cells were allowed to migrate for 4 h at 37 °C in an atmosphere of 95% air/5% CO_2_. Cells on the filter were stained with Liu’s stain and followed by washing three times with 1 × PBS. Cells on the upper surface of the filter were removed using a cotton swab. Cells on the lower surface of the filter were counted under microscope. Eight random fields (HPF) (200×) per filter and the mean number of migrating cells were calculated for each condition.

### Co-immunoprecipitation (Co-IP) analysis

The Co-IP was performed according to established method with minimal modification^21^. In brief, HUVECs were lysed in the Co-IP buffer containing 20 mM Tris-HCl (pH7.5), 100 mM NaCl, 0.5 mM EDTA, 0.5% NP-40, 0.5 mM PMSF, 0.5% protease inhibitor cocktail on ice for 20 min. The lysates were incubated with anti-eNOS antibody at 4 °C for 1 h. Dynabeads Protein G (Dynal Biotech: Invitrogen) equilibrated in Co-IP buffer was added to the solution and gently mixed for another 1 h. Insoluble immune complexes were rinsed once with Co-IP buffer then eluted by 0.1 mM citrate (pH3.0) twice for 5 min, resolved by sodium dodecyl sulfate–polyacrylamide gel, and transferred to nitrocellulose. Blots were blocked with phosphate buffered saline with 0.1% Tween 20 and then binding with antibodies for eNOS (1: 1000) or caveolin-1 (1: 1000), followed by horseradish peroxidase-conjugated goat anti-rabbit (diluted 1: 5000) and enhanced chemiluminescence reaction was performed for blots development. Band intensities were quantitated by densitometry.

### Western blot analysis

Western blotting was used to identify the presence of specific proteins in electrophoretically separated samples. The standard western blot protocol was performed as our previous study^6^. The following antibodies were used: antibodies against human eNOS, phosphor-eNOS (Ser1177), Cav-1, phosphor-Cav-1 (Tyr14), Src and phosphor-Src (Tyr416) were obtained from Cell Signaling (MA, USA). Antibody against β-tubulin was obtained from Thermo Scientific (MA, USA). The BioSpectrum Imaging System (UVP, LLC) was used to detect signal of immunopositive protein bands.

### Statistics

All results were represented as means ± standard error of the mean (SEM). The unpaired Student t test for comparison between two groups was used to determinate statistical significance. A *p* value was considered to be statistically significant as its value was less than 0.05.

## Results

### BPC 157 induced a concentration- and endothelium-dependent relaxation of isolated rat aorta

**Figure 1:**
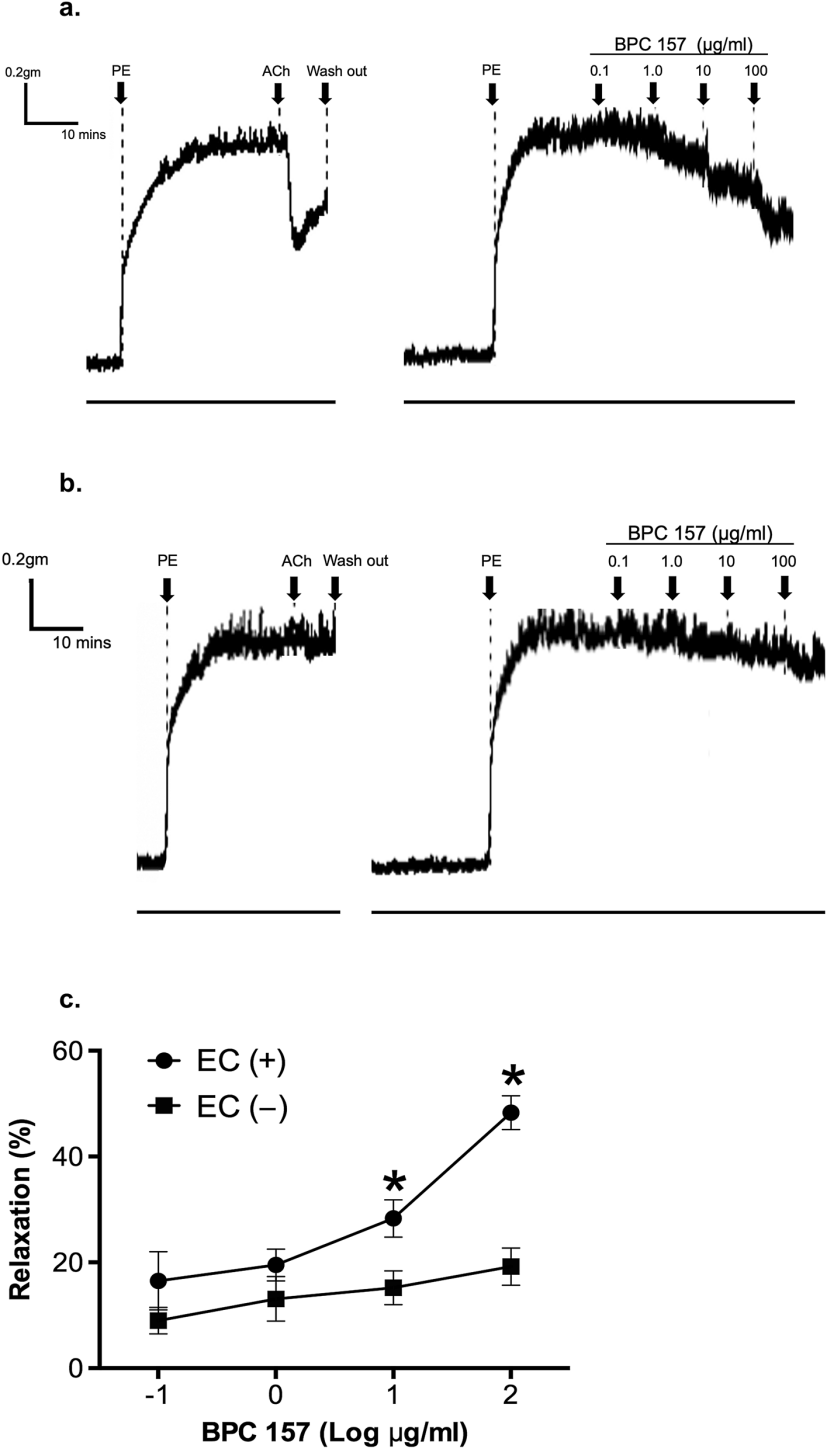
BPC 157 induced concentration- and endothelium-dependent vasorelaxation in isolated rat aortas. Representative tracking graphs of BPC 157-induced vasorelaxation in PE-preconditioned aortic rings (**a**) with intact endothelium, (**b**) without endothelium. (**c**) The percentage of maximal relaxation in each dose of BPC157 was determined and compared. Data were mean ± SEM calculated from three individual experiments (significance comparing endothelium intact or not, *p < 0.05).

The effects of the cumulative addition of 0.1–100 μg/ml BPC 157 were studied on rat aortic rings either with endothelium intact or removed. As shown in Fig. 1a, BPC 157 produced a concentration-dependent vasodilation in aortic rings with intact endothelium. The vasorelaxation induced by 0.1 or 1 μg/ml BPC 157 was 16.5 ± 5.5% or 19.5 ± 3.0%, respectively. Low concentration of BPC 157 such as 0.1 or 1 μg/ml did not induce significant vasorelaxation. However, following the cumulative addition of BPC 157 up to 10 or 100 μg/ml, the vasorelaxation increased to 28.3 ± 3.5% or 48.3 ± 3.2%, respectively. The vasodilation response induced by BPC 157 appeared immediately after the treatment and reached a maximum value 5 min after. This vasodilation response was sustained and continued without further change till the end of observation.

In aorta rings without endothelium, the vascular relaxation induced by BPC 157 was markedly attenuated (Fig. 1b). The induction of vasorelaxation in aorta without endothelium by 0.1, 1, 10, or 100 μg/ml BPC 157 was 9.0 ± 2.5%, 13.1 ± 4.2%, 15.2 ± 3.2% or 19.2 ± 3.5%, respectively. There was no significant difference in vasorelaxation induced by 0.1 or 1 μg/ml BPC 157 in aorta without endothelium. Following the cumulative addition of BPC 157 at high concentration such as 10 or 100 μg/ml, a slightly increased vasorelaxation in aorta without endothelium was noticed which deserved further investigation (Fig. 1c). Overall, these findings indicated that BPC 157 exerted a concentration- and endothelium-dependent effect on the relaxation of large vessels.

### BPC 157 induced the vasorelaxation via eNOS/nitric oxide singling activation

**Figure 2:**
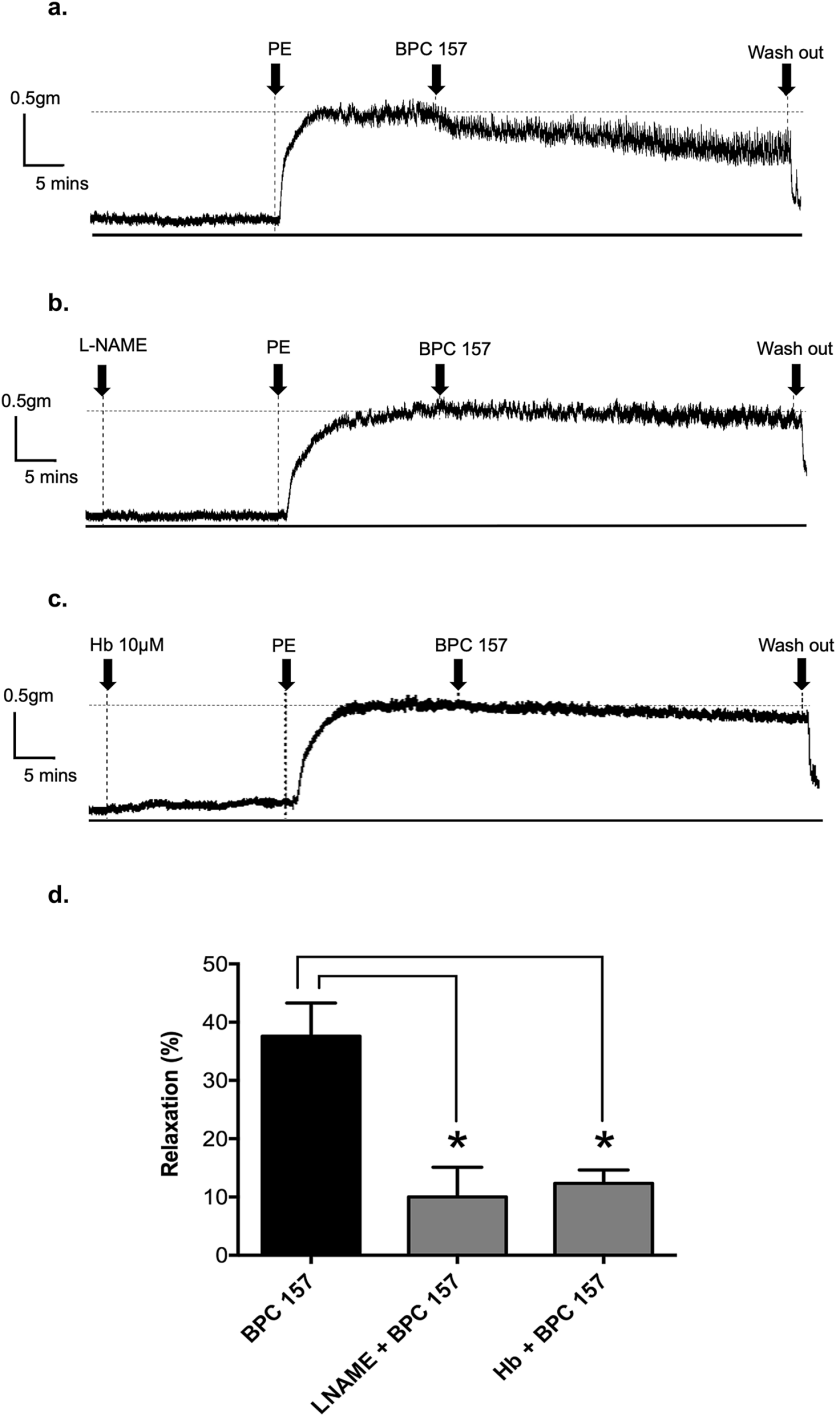
BPC 157 induced a NO-dependent vasorelaxation in isolated rat aortas. Representative tracking graphs of BPC 157 (100 μg/ml)-induced vasorelaxation in PE-preconditioned aortic rings in the presence of (**a**) vehicle, (**b**) 0.03 mM L-NAME, (**c**) 10 μM hemoglobin. (**d**) The percentage of vasorelaxation in each condition was determined and compared. Data were mean ± SEM calculated from three independent experiments (significance compared with vehicle, *p < 0.05).

In order to investigate whether BPC 157 induced vasorelaxation via eNOS-nitric oxide singling activation, L-NAME (eNOS inhibitor) and hemoglobin (nitric oxide scavenger) were used in further studies. Following a single addition of 100 μg/ml BPC 157, the vasorelaxation reached 37.6 ± 5.7% (Fig. 2a). In the presence of 0.03 mM L-NAME, the maximal vasorelaxation induced by 100 μg/ml BPC 157 was decreased to 10.0 ± 5.1% (Fig. 2b). In the presence with 10 μM hemoglobin, vasorelaxation induced by 100 μg/ml BPC 157 was decreased to 12.3 ± 2.3% (Fig. 2c). Together, these results demonstrated the important role of eNOS/nitric oxide activation in the BPC 157 effect on blood vessel (Fig. 2d).

### No direct effect of BPC 157 on the relaxation of vascular smooth muscle cells in 3-D model

**Figure 3:**
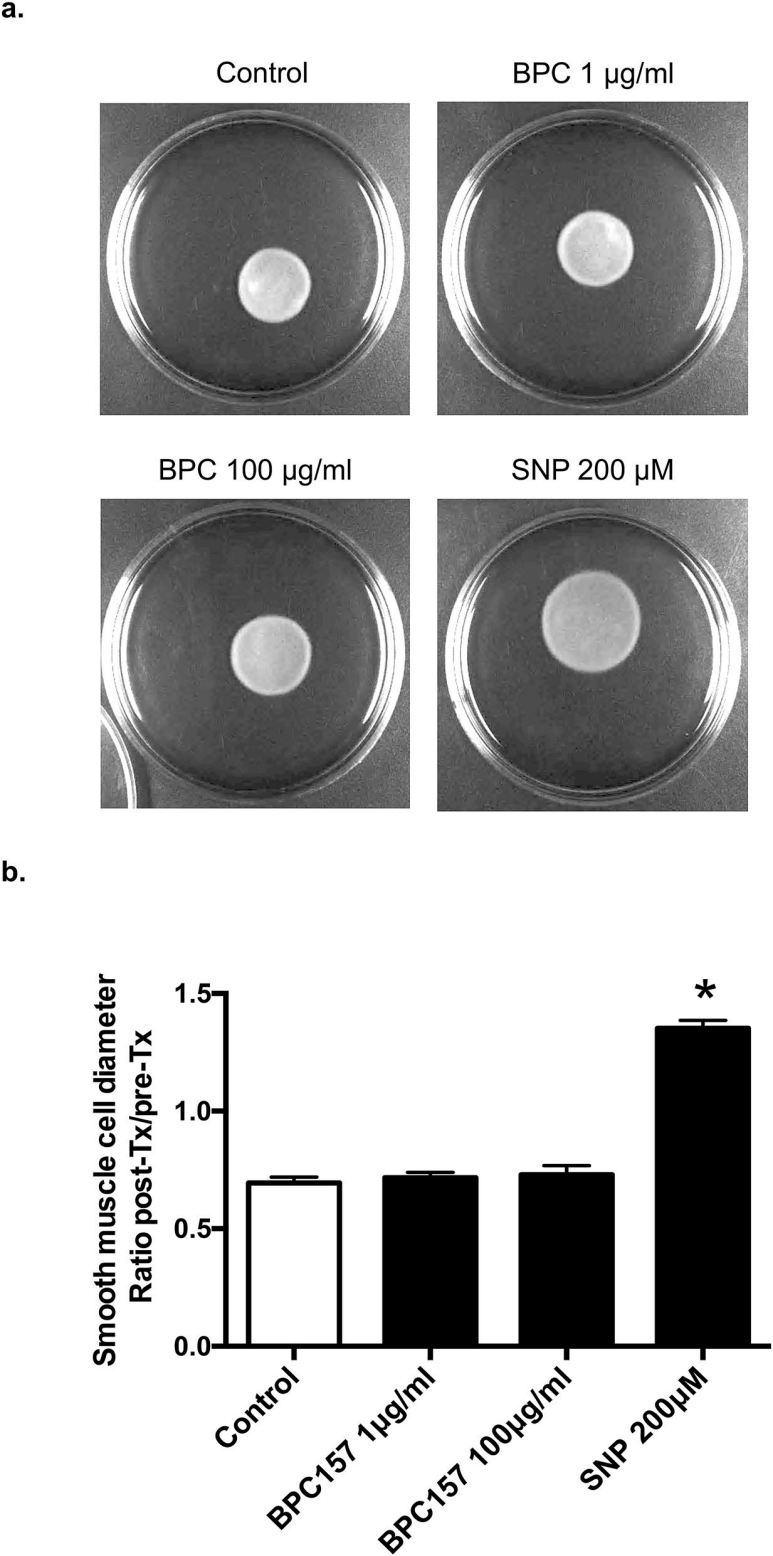
BPC 157 did not induce the relaxation of vascular smooth muscle cells in 3-D models. Representative photos of vascular smooth muscle cells in 3D-models treated with (**a**) vesicle, 1 μg/ml BPC 157, 100 μg/ml BPC 157 and 200 μM SNP, (**b**) representative data showed the ratio comparing the diameters of 3D-cell disc before and after treatment in 4 groups (*means compared with control *p* value < 0.05).

In order to investigate whether BPC 157 had relaxation effect on vascular smooth muscle cells, a 3-D model was used. 3-D cell discs were formed by culturing vascular smooth muscle cells in collagen first and then 3-D discs with similar size were incubated in different media for 24 h. The ratio of diameter change compared with the original size was 0.69 ± 0.02, 0.72 ± 0.02, 0.73 ± 0.04, and 1.35 ± 0.03 in control, 1.0 μg/ml BPC 157, 100 μg/ml BPC 157 and 200 μM SNP, respectively (Fig. 3a,b). Compared with control, the nitric oxide donor SNP resulted in the dilation of 3-D cell disc significantly. However, BPC 157 at low or high concentration did not cause any change of disc diameter as compared to control. This result indicated that BPC 157 could not induce the relaxation directly on the 3-D model composed of vascular smooth muscle cells, likely due to the lack of nitric oxide stimulation. It is therefore suggesting that the slight change of relaxation observed in the aorta without endothelium ex vivo might be due to some mechanism unrelated to the direct nitric oxide stimulation which needs further investigation.

### Nitric oxide induced by BPC 157 contributed to the promoted cell migration

**Figure 4:**
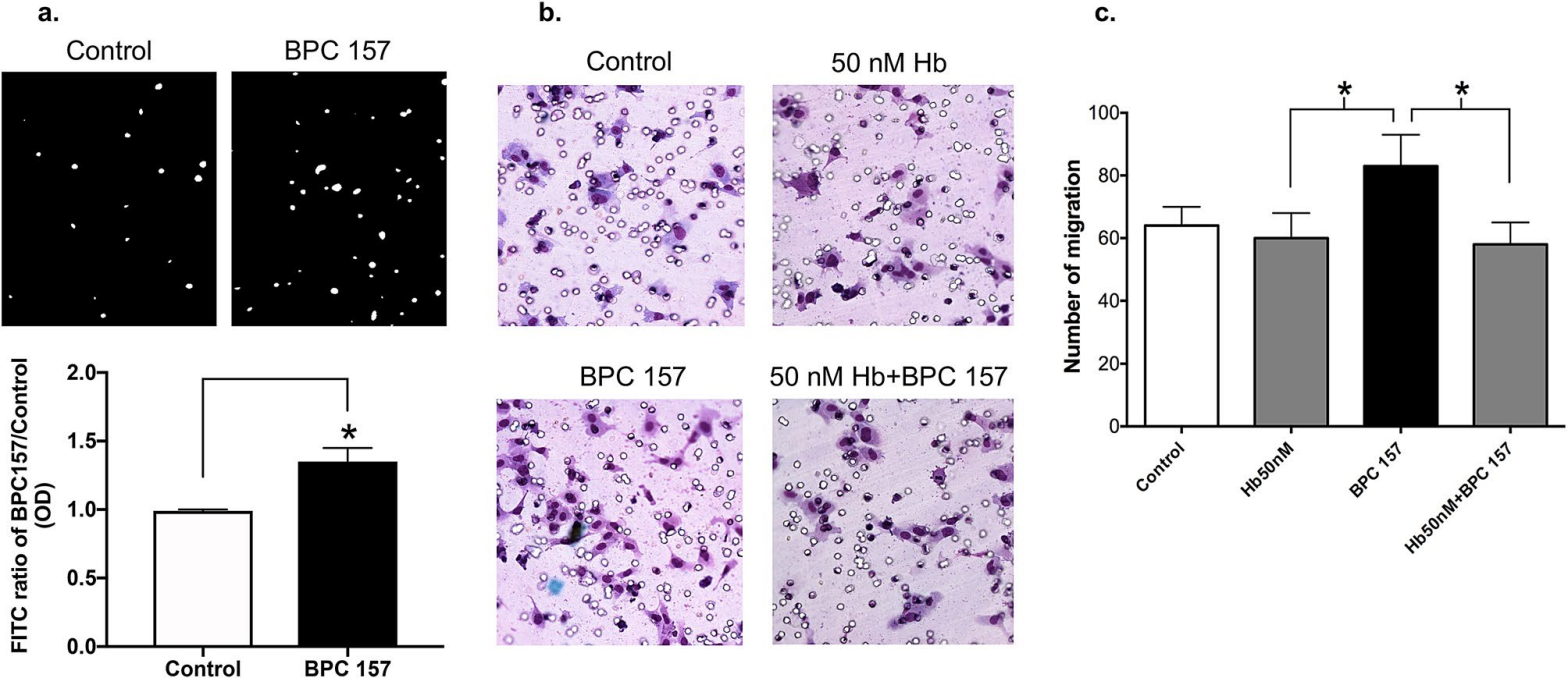
Nitric oxide induced by BPC 157 contributed to the promoted cell migration. (**a**) HUVECs were stimulated with 1 μg/ml BPC 157, then the amount of nitric oxide production was assessed by the fluorescence (white spots shown in upper panel) generated by DAF-FM DA fluorescent probe. Results were calculated and shown in lower panel (*means compared with control *p* value < 0.05), (**b**) Representative photos showing the migration of HUVECs treated with vehicle, 1 μg/ml BPC 157, 50 nM hemoglobin and 1 μg/BPC 157 together with 50 nM hemoglobin. (**c**) Compared with vehicle, the number of cell migration in each group was determined. Data were shown as mean ± SEM of three independent experiments (*means compared in two groups, *p* value < 0.05).

The in vitro angiogenic effect of BPC 157 has been shown to promote the cell migration of vascular endothelial cells in our previous study. Nitric oxide production is known to play an important role on mediating angiogenesis^26^. We then measured the effect of BPC 157 on nitric oxide production in vascular endothelial cells and examined the role of nitric oxide in BPC 157-induced cell migration. Cellular nitric oxide production in vascular endothelial cells was detected by the emitted fluorescence from DAF-FM DA probe upon the interaction with nitric oxide. Result in Fig. 4a indicated that BPC 157 at 1 μg/ml could increase the nitric oxide production in vascular endothelial cells up to 1.35 ± 0.1-fold compared with control. In Fig. 4b, cell migration enhanced by BPC 157 was completely suppressed in the presence of hemoglobin since nitric oxide were chelated (Fig. 4c).

### BPC 157 activated the Src-Cav-1-eNOS signal pathway and reduced eNOS/Cav-1 binding in vascular endothelial cells

**Figure 5:**
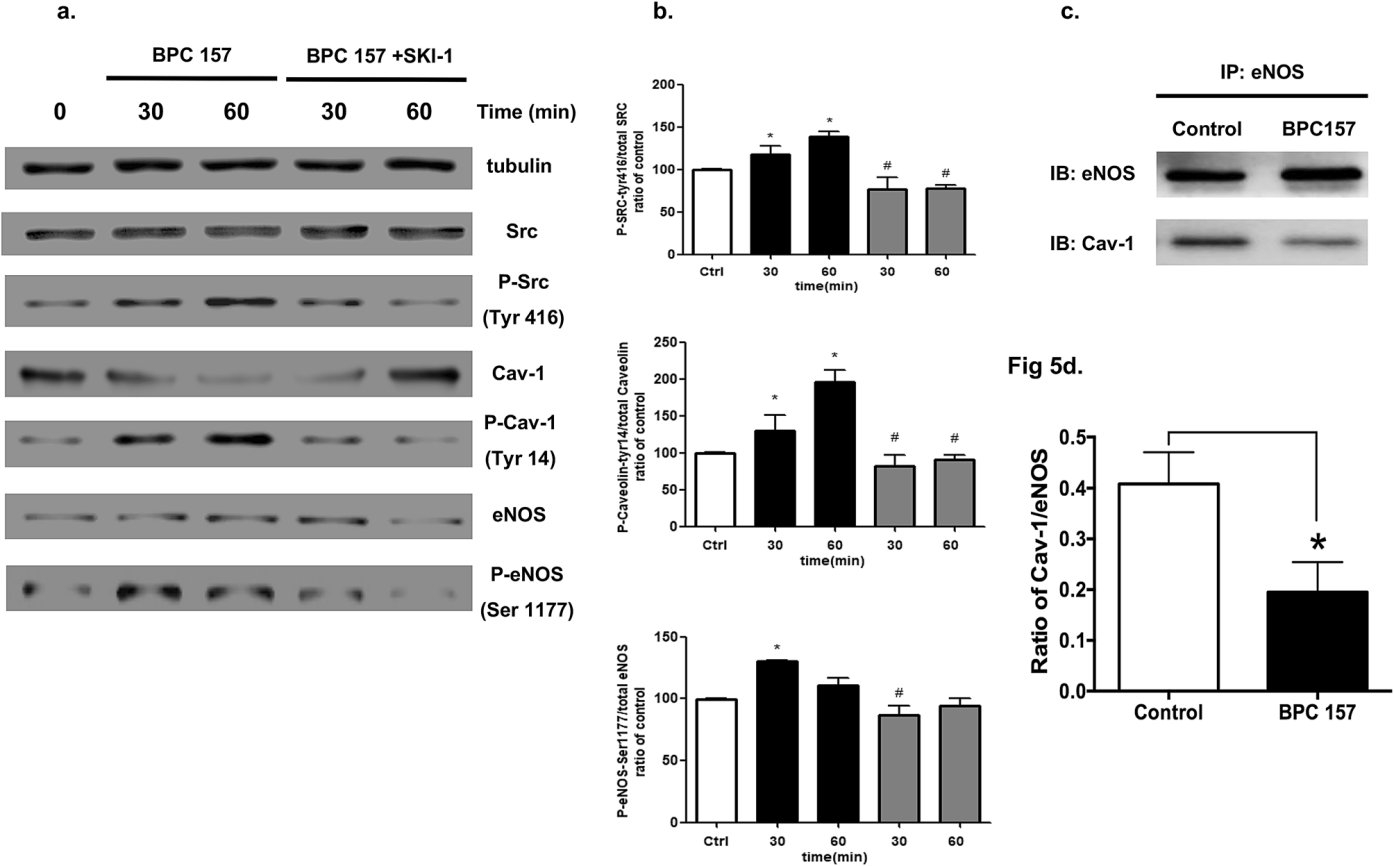
BPC 157 activated the Src-Cav-1-eNOS signaling pathway and reduced Cav-1-eNOS interaction. HUVECs were stimulated with 1 μg/ml BPC 157 for different time periods as indicated and the phosphorylation of Src (Tyr 416), Cav-1 (Tyr14) and eNOS (Ser 1177) was analyzed by western blot. Representative blots were shown in (**a**) and bands were scanned and quantified by densitometric analysis in (**b**). Data were shown as mean ± SEM of three independent experiments (*means compared with control, *p* value < 0.05; #means BPC 157 + SKI-1 compared with BPC 157 at same treatment period, *p* value < 0.05). The Co-IP was also used to assess the interaction between Cav-1 and eNOS. The representative blot and band quantified by densitometric analysis were shown in (**c**) and (**d**) respectively (*means compared with control *p* value < 0.05).

It is known that phosphorylation of Src can promote Cav-1 activation and subsequent endocytosis of tyrosine kinase receptors and eNOS activation^20,24^. The increase of eNOS phosphorylation upon stimulated with BPC 157 has been proved in our previous study^6^. Therefore, in this study we addressed whether BPC 157 could activate the Src-Cav-1-eNOS signal pathway. HUVECs were treated with 1.0 μg/ml BPC 157 and the protein expressions of Src, Cav-1, eNOS and their phosphorylated forms were analyzed by western blot. Results shown in Fig. 5a demonstrated that BPC 157 could induce a quick increase of Src phosphorylation at 30 and 60 min with the amount of total Src remained constant. Similar change of pCav-1 was found, however, the total amount of Cav-1 was decreased. The phosphorylation of eNOS was also increased and peaked at 30 min after the BPC 157 stimulation. Also shown in Fig. 5a, the pretreatment with Src inhibitor (SKI-1) significantly reduced the effect of BPC 157 on the phosphorylation of Src, Cav-1 and eNOS, further confirming the critical upstream role of Src in this pathway. Digital scanning analysis of these protein bands was shown in Fig. 5b. The activity of eNOS is known to be regulated by its binding with Cav-1. Increased eNOS activity is associated with the reduced binding to Cav-1^17,18^. BPC 157 increased nitric oxide production and activated the eNOS phosphorylation as reported in our previous study^6^. We therefore studied whether BPC 157 could modulate the interaction between eNOS and Cav-1. Results from CO-IP demonstrated that 1.0 μg/ml BPC 157 decreased the eNOS/Cav-1 binding to 50% relative to vehicle-treated control (Fig. 5c,d). Decrease of Cav-1 binding released the eNOS and subsequently enhanced the activation of eNOS.

## Discussion

The major findings of this study are as follows: (1) BPC 157 induces an ex vivo nitric oxide-mediated relaxation of large vessel in a concentration-dependent manner. (2) BPC 157 promotes the vessel relaxation mainly acting on vascular endothelial cells but much less on vascular smooth muscle cells. (3) BPC 157 increases the nitric oxide production that contributes to the migration of vascular endothelial cells. (4) BPC 157 activates the signal pathways of Src, Cav-1 and eNOS. (5) BPC 157 reduces the protein–protein interaction between eNOS and Cav-1.

Under ex vivo condition, BPC 157 induced a concentration-dependent vasodilation in intact aortic rings. Low concentration of BPC 157 at 0.1 or 1.0 μg/ml did not induce significant vasorelaxation. Only when BPC 157 was cumulatively added up to 10 or 100 μg/ml, the increase of vasorelaxation became prominent. Our previous study reported that the treatment of 10 μg/Kg/day BPC 157 could accelerate the recovery of blood flow in the ischemic muscle of the rat hind limb as detected by laser Doppler scanning. The effect was due to accelerated angiogenesis but not vasodilation, since there was no significant difference of the blood flow in tails and non-ischemic hind limb between the control and BPC 157 groups. In addition, the blood pressure of tails was not significantly different between the control and BPC 157 groups either^5,6^. In rat with the treatment of 10 μg/Kg/day BPC 157, the concentration of BPC 157 was estimated to be less than 1 μg/ml in the blood which was too low to cause hypotension or hemodynamic change as demonstrated by the lack of vasodilation in the present study^6^. However, BPC 157 is relatively stable and small dose ranged between ng-μg/ml or ng-μg/kg is already effective on promoting tissue injury healing and angiogenesis in many animal studies^4,27–30^.

BPC 157 has been demonstrated to reverse the hypertension induced in hyperkalemic rat^31^. With BPC 157 therapy, portal hypertension in bile duct ligation-rats is either not even developed or rapidly abated^32^. BPC 157 has also been found to counteract both vein hypertension and arterial hypotension in rat interior vein ligature model^33^. The modulatory effect of BPC 157 on balancing the nitric oxide-related system including counteracting L-NAME-hypertension as well as L-arginine-hypotension in different animal studies have been largely reported^2,7,8,34^. In this study, although we proved that ex vivo nitric oxide- and endothelium-related vasodilation in normal large vessel was induced only by BPC 157 at high concentration, it could not be ruled out that BPC 157 at low concentration might counteract the imbalanced blood pressure under an in vivo diseased condition through a currently unknown mechanism. Study using DAF-FM fluorescent dye revealed a 1.35-fold increase of nitric oxide induced in vascular endothelial cells by 1.0 μg/ml BPC 157 which contributed to the enhanced cell migration. Hemoglobin, as an effective nitric oxide chelator, consumes one nitric oxide by binding to each heme group as demonstrated in Donadee’s equation^35^. The BPC 157-enhanced cell migration was completely suppressed in the presence of 50 nM hemoglobin, suggesting that the amount of nitric oxide produced by 1.0 μg/ml BPC 157 did not exceed 200 nM because hemoglobin contains four heme groups in each molecule. The well-known healing effect of BPC 157 occurring at low level of nitric oxide might potentially imply a more clinical utilization without toxicity relative to excessive nitric oxide^36,37^.

Cav-1 is crucial in maintaining vascular health but a paradoxical role of Cav-1 in cardiovascular pathophysiology has also been reported^19,38^. Direct binding of eNOS to the scaffolding domain of Cav-1 is a well-accepted mechanism to repress eNOS activity that is associated with endothelial dysfunction and cardiovascular disease^39,40^. Many cardiovascular risk factors such as diabetes mellitus, hypertension and hyperlipidemia are demonstrated to stimulate the interaction of Cav-1 and eNOS, reduce nitric oxide production resulting in endothelial dysfunction and subsequently the formation of atherosclerotic lesion^41–43^. Conversely, decreased Cav-1 level or obstructed Cav-1 and eNOS interaction can release eNOS favoring its activation and induction of nitric oxide production that is known for its protective effect on vessels^44,45^. The present study, for the first time, demonstrates an underlying mechanism for the activation of eNOS by BPC 157 on stimulating the Cav-1 phosphorylation and reducing the binding of eNOS to Cav-1.

In addition to the regulation of eNOS activity, Cav-1 also can modulate the activation of VEGFR2. It has been shown that VEGFR2 is associated with Cav-1 and located in endothelial caveolae. The phosphorylation of Src and Cav-1 is an important process that mediates the endocytosis of VEGFR2 and triggers the eNOS activation in vascular endothelial cells^19,20^. BPC 157 can enhance the expression and endocytosis of VEGFR2, and subsequently the phosphorylation of AKT and eNOS as proved in our previous study^6^. In the present study, we further demonstrate that BPC 157 can promote the phosphorylation of Src and Cav-1, providing a strong explanation for the activation and endocytosis of VEGFR2 by BPC 157 as reported in our previous study. In conclusion, as shown in Fig S1, the activation of eNOS by BPC 157 is mediated through multiple regulation processes including the promotion of VEGFR2 activation and endocytosis, the activation of positive regulator AKT, the reduction of the interaction of negative regulator Cav-1 with eNOS. BPC157 modulated multiple intracellular signal pathways to enhance its clinical application potential^46^.

## Supplementary information

Supplementary Information 1.

Supplementary Information 2.
