# Supplementary material for: Modulatory effects of BPC 157 on vasomotor tone and the activation of Src-Caveolin-1-endothelial nitric oxide synthase pathway
Source: Sci Rep. 2020 Oct 13;10:17078. doi: 10.1038/s41598-020-74022-y (PMC7555539; doi:10.1038/s41598-020-74022-y)

Modulatory effects of BPC 157 on vasomotor tone and the activation of Src-Caveolin-1-endothelial nitric oxide synthase pathway

Ming-Jer Hsieh[1, 2], M.D, PHD; Cheng-Hung Lee [2], M.D, PHD; Ho-Yen Chueh[3], M.D; Gwo-Jyh Chang[1], PHD; Hsiu-Yun Huang[1], M.S; Yuling Lin[1], M.S; Jong-Hwei S. Pang[1,4], PHD

[1] Graduate Institute of Clinical Medical Sciences, Chang Gung University, College of Medicine, Tao-Yuan City, Taiwan, ROC.

[2] Division of Cardiology, Department of Internal Medicine, Chang Gung Memorial Hospital-Linkou, Chang Gung University, Tao-Yuan City, Taiwan, ROC.

[3] Department of Obstetrics and Gynecology, Chang Gung Memorial Hospital, Lin-Kou Medical Center, Tao-Yuan City, Taiwan, ROC.

[4] Department of Physical Medicine and Rehabilitation, Chang Gung Memorial Hospital, Kwei-Shan,Tao-Yuan City, Taiwan, ROC.

Fig. S1

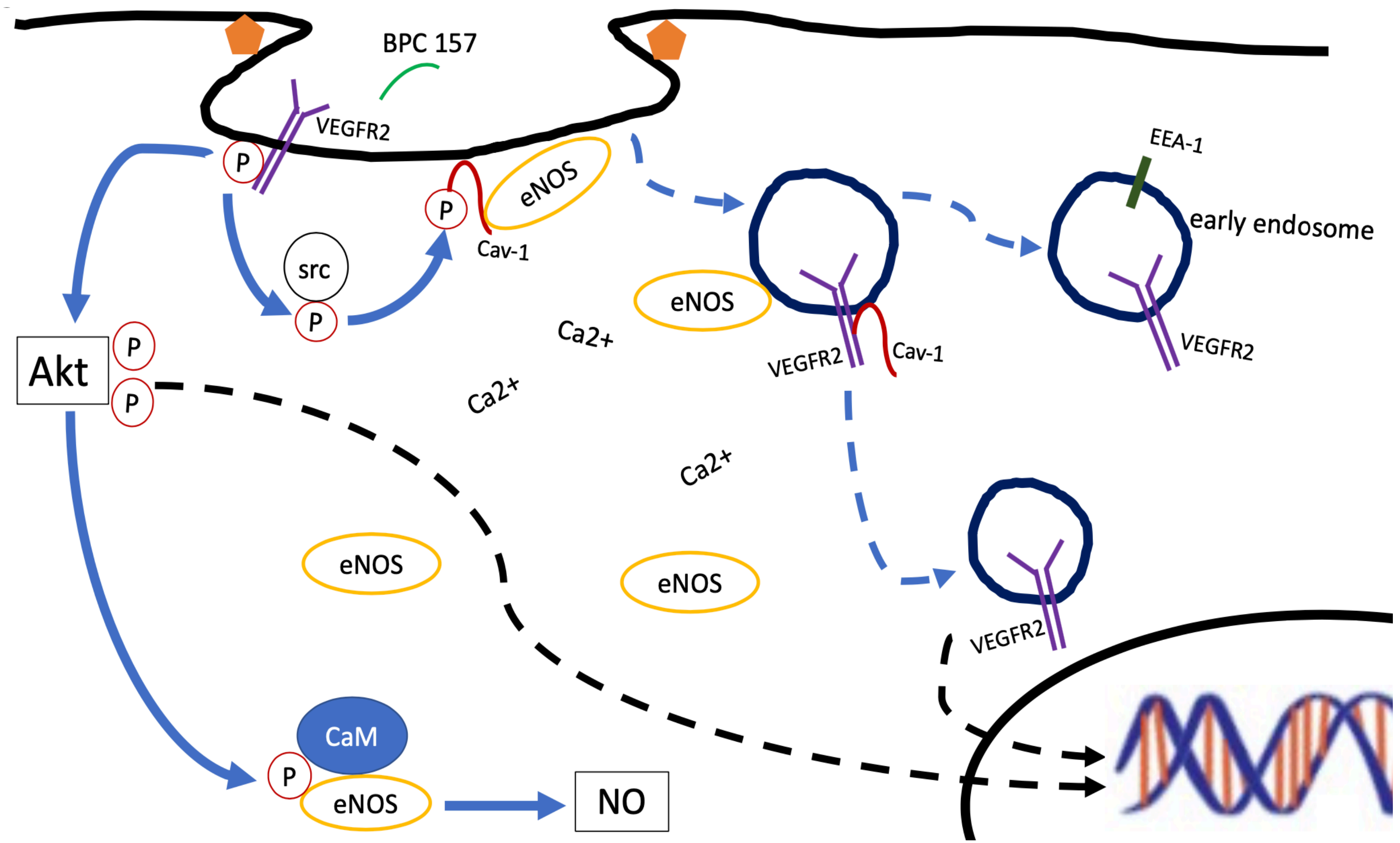
BPC 157
VEGFR2
P
eNOS
Cav-1
src
P
P
Akt
P
P
eNOS
Ca2+
Ca2+
Ca2+
VEGFR2
Cav-1
EEA-1
early endosome
VEGFR2
eNOS
eNOS
VEGFR2
CaM
P
eNOS
NO

Fig 5a. Supplement

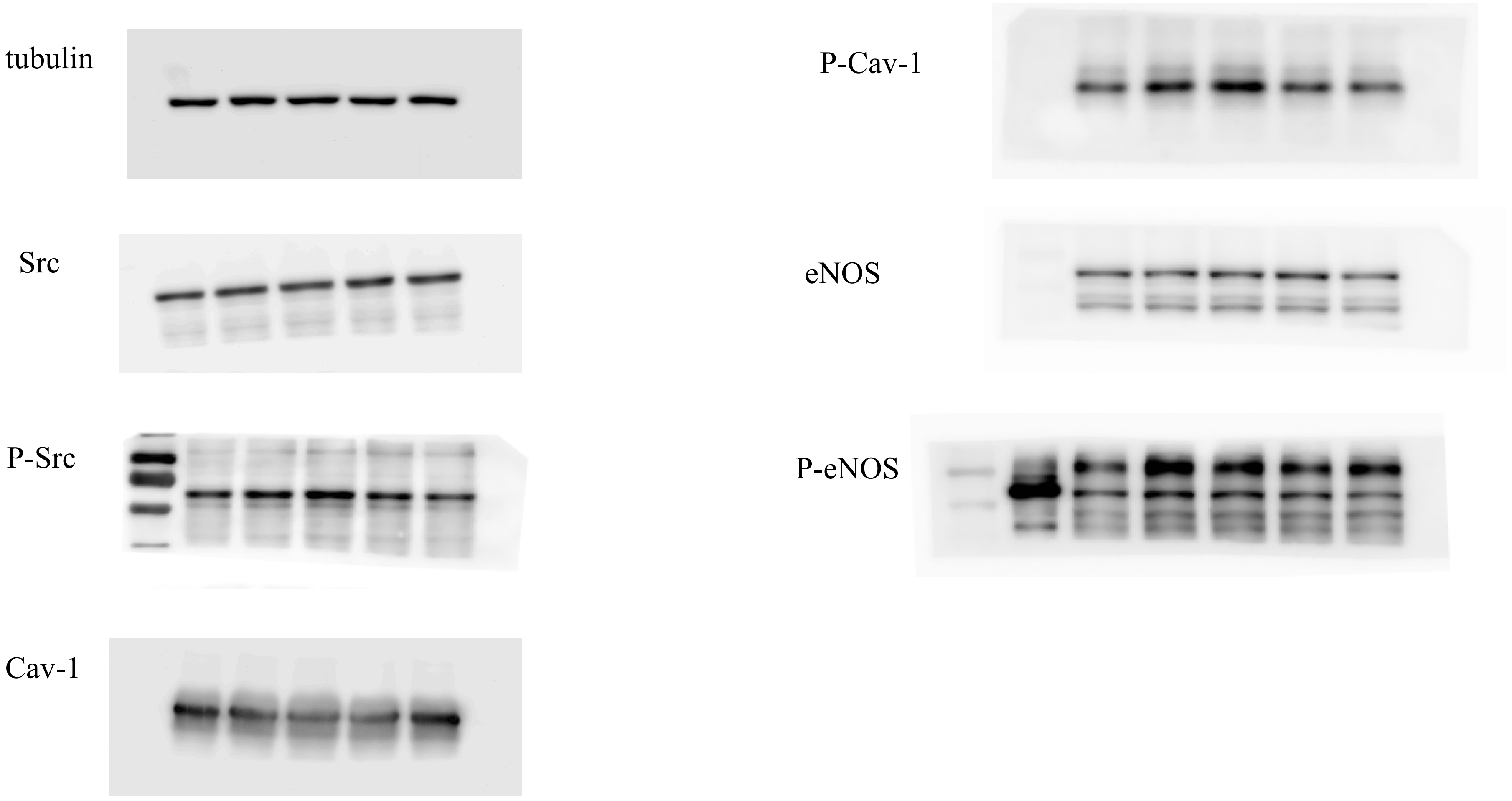

Fig 5c. Supplement

IB: eNOS

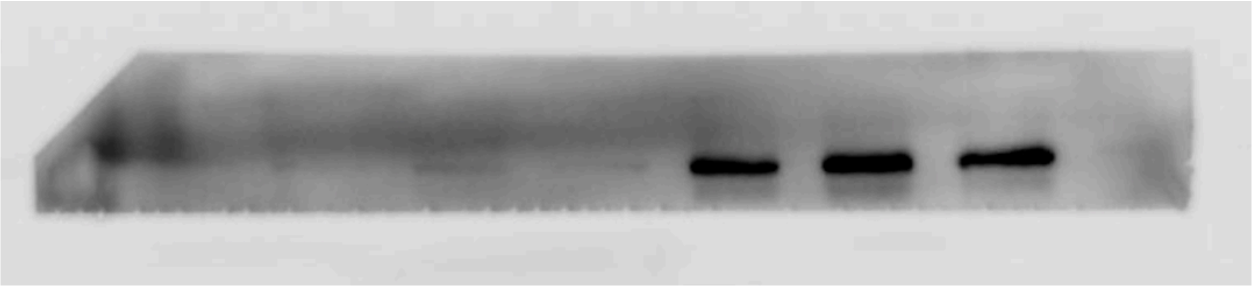

IB: Cav-1

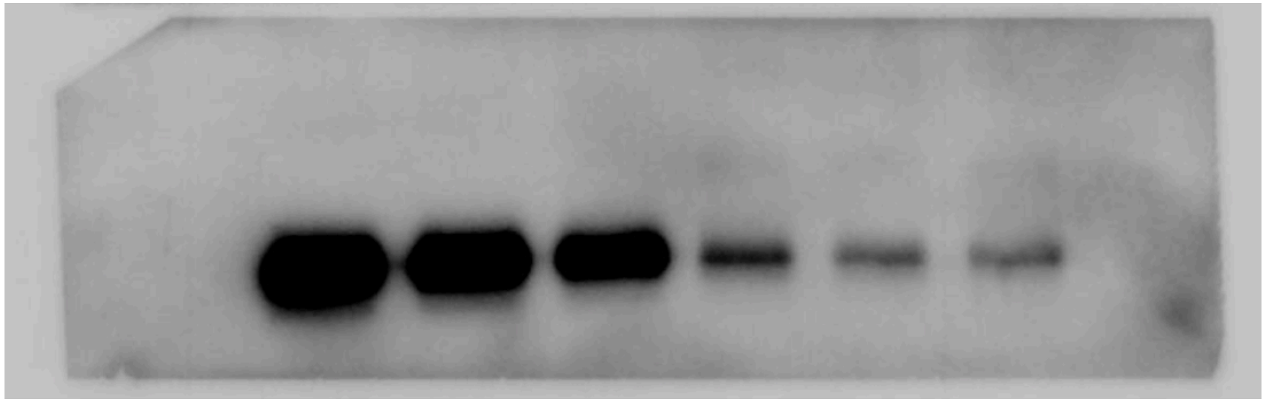

Supplement: Supplementary file 1 — Supplementary Information 1. [file 41598_2020_74022_MOESM1_ESM.pdf]
