# Supplementary material for: Modulatory effects of BPC 157 on vasomotor tone and the activation of Src-Caveolin-1-endothelial nitric oxide synthase pathway
Source: Sci Rep. 2020 Oct 13;10:17078. doi: 10.1038/s41598-020-74022-y (PMC7555539; doi:10.1038/s41598-020-74022-y)
Supplement: Supplementary file 2 — Supplementary Information 2. [file 41598_2020_74022_MOESM2_ESM.docx]

**Figure S1. BPC 157 activates eNOS through multiple intracellular regulatory pathways in vascular endothelial cells**. VEGFR2 activation and endocytosis, AKT activation, phosphorylation of Src and Cav-1, and reduced Cav-1 interaction with eNOS all contribute to the eNOS activation and following nitric oxide production.
